# Optimisation of Substrate Angles for Multi-material and Multi-functional Inkjet Printing

**DOI:** 10.1038/s41598-018-27311-6

**Published:** 2018-06-13

**Authors:** Jayasheelan Vaithilingam, Ehab Saleh, Ricky D. Wildman, Richard J. M. Hague, Christopher J. Tuck

**Affiliations:** Centre for Additive Manufacturing, Faculty of Engineering, University of Nottingham, Nottingham, NG7 2RD UK

## Abstract

Three dimensional inkjet printing of multiple materials for electronics applications are challenging due to the limited material availability, inconsistencies in layer thickness between dissimilar materials and the need to expose the printed tracks of metal nanoparticles to temperature above 100 °C for sintering. It is envisaged that instead of printing a dielectric and a conductive material on the same plane, by printing conductive tracks on an angled dielectric surface, the required number of silver layers and consequently, the exposure of the polymer to high temperature and the build time of the component can be significantly reduced. Conductive tracks printed with a fixed print height (FH) showed significantly better resolution for all angles than the fixed slope (FS) sample where the print height varied to maintain the slope length. The electrical resistance of the tracks remained under 10Ω up to 60° for FH; whereas for the FS samples, the resistance remained under 10Ω for samples up to 45°. Thus by fixing the print height to 4 mm, precise tracks with low resistance can be printed at substrate angles up to 60°. By adopting this approach, the build height “Z” can be quickly attained with less exposure of the polymer to high temperature.

## Introduction

Additive Manufacturing (AM), also referred to as three dimensional (3D) printing has shown the capability to produce parts with customised and complex designs and is being embraced by various industrial sectors. Recently, an aspiration has been to include both structural and functional elements to realise *multifunctional* AM (MFAM) parts^1^. This will enable a single component to perform both structural and electrical (or other) functions, for example, actuators embedded in a moving component, heating elements incorporated to a component to alter its temperature or the integration of strain gauges to monitor the performance of a component^2^. Thus the interest for MFAM, is increasing since it has the potential to produce novel and high-value parts with multiple functionality^3,4^.

Inkjet Printing (IJP) is a material jetting based AM technique that jets droplets of inks either continuously or using a drop-on-demand (DoD) onto a build platform to additively create a 3D structure. By supplying different inks to each printhead and exposing the printed layers to a ultra-violet or a heat source for consolidation, over successive layers, multi-material 3D components can be created and hence, IJP is considered as one of the key enablers of MFAM^4,5^. Previous studies have also demonstrated the fabrication of multifunctional components using inkjet based printing systems for various mechanical, electrical and electronics applications^6–11^. However in these studies, realisation of multi-functionality was not achieved in a single step; rather the parts had to be typically transferred from one printing system to another to fabricate/assemble dielectrics and conductive elements to make the component fully functional.

Although IJP has the capability to print multiple materials, fabricating a 3D multi-functional component with conductive routing in a single step is still a challenge. Challenges that constrain one-step MFAM include: limited availability of materials for printing; the lack of equipment that can both print and consolidate multiple materials in multiple ways; design challenges; inconsistencies in the layer thickness of different materials; thermal and chemical stability of printed materials; and surface/chemical interaction between dissimilar materials. Among these issues, this study is focussed on addressing the layer thickness inconsistencies between dissimilar materials and reducing its impact on the 3D MFAM for electronics applications.

The thickness of a single printed layer depends on various parameters such as the viscosity of the ink, the volume of droplet and the solid content of the ink. Inks made of silver nanoparticles (AgNPs) are widely used to inkjet print conductive circuits and among polymers, polyimides are preferred as a dielectric material due to its high thermal stability. When these materials are co-printed and consolidated, the thickness of the polymer film is at least 1 order of magnitude higher than the thickness of the silver layer^12–14^. This difference is due to the high loading of the photopolymers with almost no solvent as oppose to the ink with AgNPs where the silver content of the ink is ~40% and the remainder is mainly the solvent that evaporates during the sintering process. Hence, in order to 3D Print a part with embedded circuitry in the ‘Z’ (layer-stacking) plane, multiple layers of conductive material have to be printed. In addition to the time that it would take to print several layers of conductive material, every printed layer of AgNPs requires post-process sintering (typically above 130 °C) for a few minutes to achieve functionality^5,15,16^. Although AgNPs can be sintered using various sources such as microwave, ultra-violet radiation, plasma, laser and chemically-induced sintering, thermal sintering is most favoured since it is relatively less expensive and it can be easily incorporated into the printing system^4,17,18^. However, the exposure of polymers to high temperatures will not only affect their thermal stability, but it will also lead thermal expansion. As a consequence, a build comprising multiple functional/structural materials will expand at different rates when exposed to heat and this may have an implication on the fabricated part in terms of accuracy and may even lead to crack propagation or fracture. Although increasing the silver content in AgNP ink can be a possible solution to increase the layer thickness, there are significant challenges in achieving this due to the rheological constraints for IJP printing.

**Figure 1:**
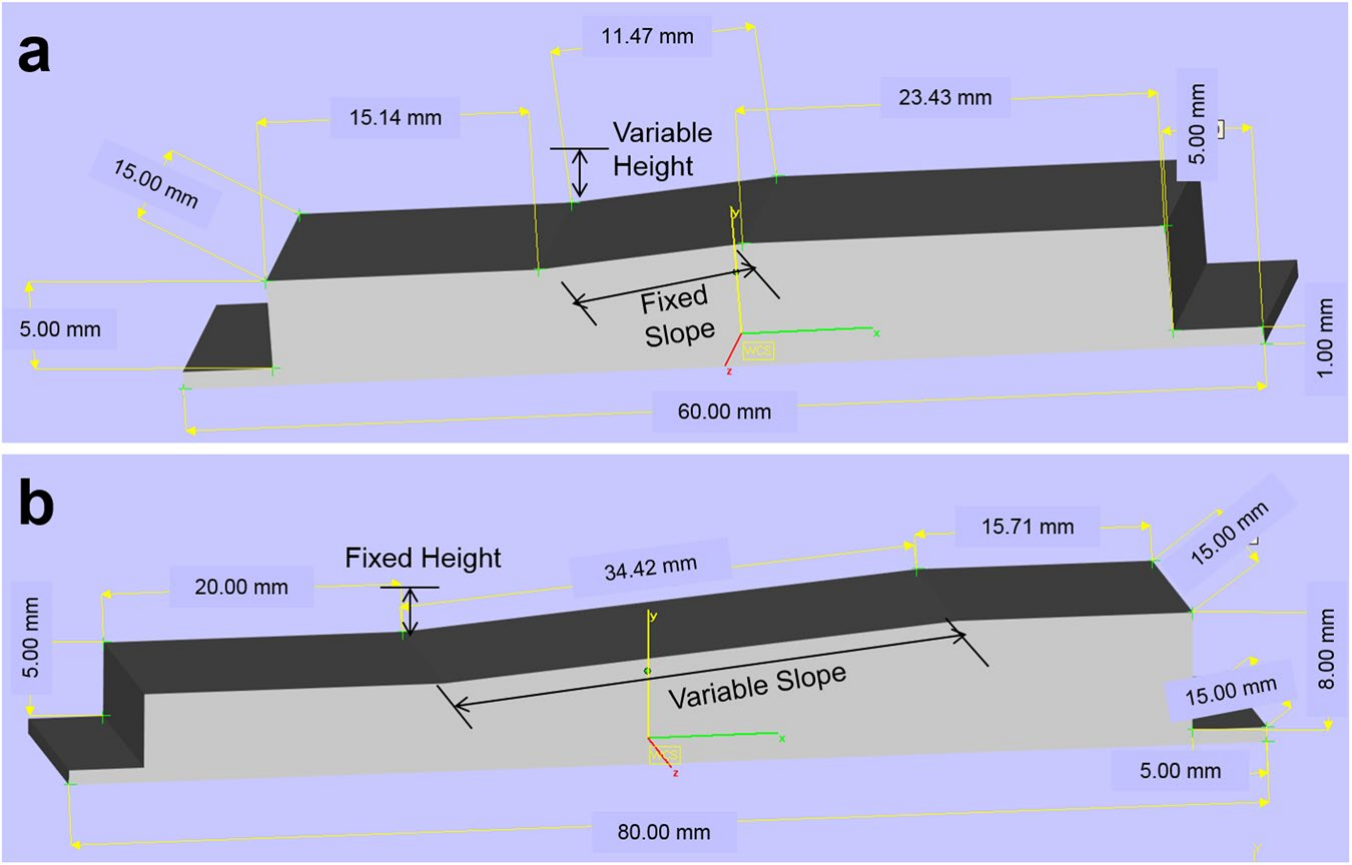
Dimensions of fixed slope – FS **(a)** and fixed height **(b)** samples.

It this study, to address the layer inconsistency issue and reduce the time of exposure of the dielectric polymer material to high temperatures from the in-process consolidation of the AgNPs, instead of printing multiple layers of conductive tracks in plane with the polymeric material, the conductive inks are printed on an angular polymeric surface. Our previous study on optimising the substrate angle was performed on substrates with fixed slope and varied print height to attain the required angle^19^. However, after a 3 mm print height, the jetted droplets were observed to scatter around the tracks and affect the print resolution and in turn the electrical resistance. It is envisaged that by printing from a fixed print height, fully functional and tracks with better resolution can be obtained at steeper angles than that reported previously. Hence in this study, a fixed print height approach (varying slope distance) was investigated and compared with the fixed slope approach (Fig. 1).

Although JETx is capable of processing multiple materials^4^, printing multiple samples for this study is time consuming due to the limited availability of print nozzles (128) and reservoir capacity to hold the ink (4 mL). As the focus of this study is to optimise the substrate angle, the dielectric surfaces with varied angles were printed using Objet Connex3 260′. The ideal print height and the substrate angle to which the AgNP ink can be printed have been optimised and presented. To further extend the scope of this work, substrates angles from 0° to 80° with 5° increments were studied for both sample sets and the effect of thermal properties of the polymer on the conductive tracks were also reported.

## Results and Discussion

### Droplet characterisation

**Figure 2:**
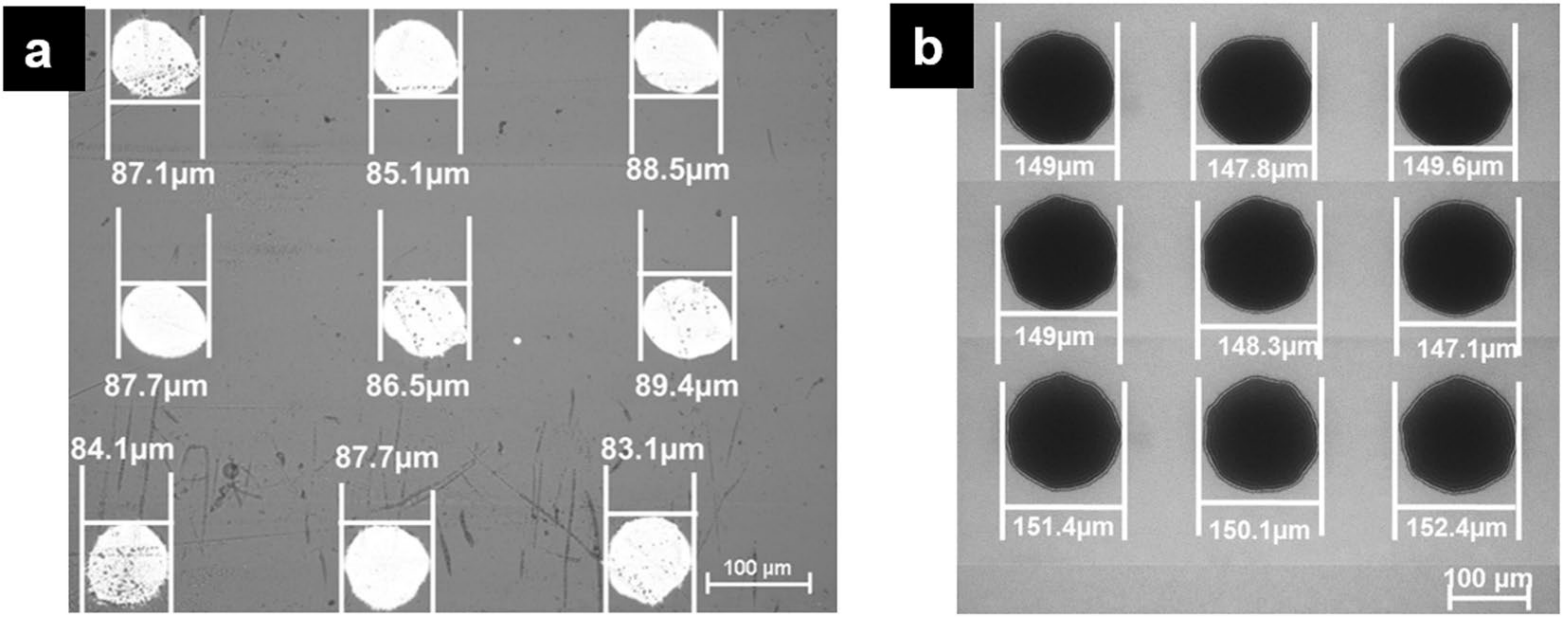
AgNP ink droplets formed on **(a)** Veroclear surface and **(b)** silver surface.

Initially, five layers of AgNPs were printed and sintered on a glass surface to characterise AgNP droplets formed on this silver surface. Droplets of AgNP ink printed on a VeroClear substrate and a silver surface was obtained using an optical microscope and shown in Fig. 2. Silver surface was obtained by printing and sintering 2 layers of AgNP ink on the glass surface. The results revealed that the size of the droplets formed on the VeroClear (87.1 ± 1.6 µm) was 40% smaller than the droplets on silver surface (149.2 ± 1.8 µm). This significant difference in the droplet size between the VeroClear and the silver surface are due to the different wetting behaviour of the AgNP ink on these surfaces. Similar wetting behaviour has been previously observed^20^.

### Surface topography of VeroClear substrate

**Figure 3:**
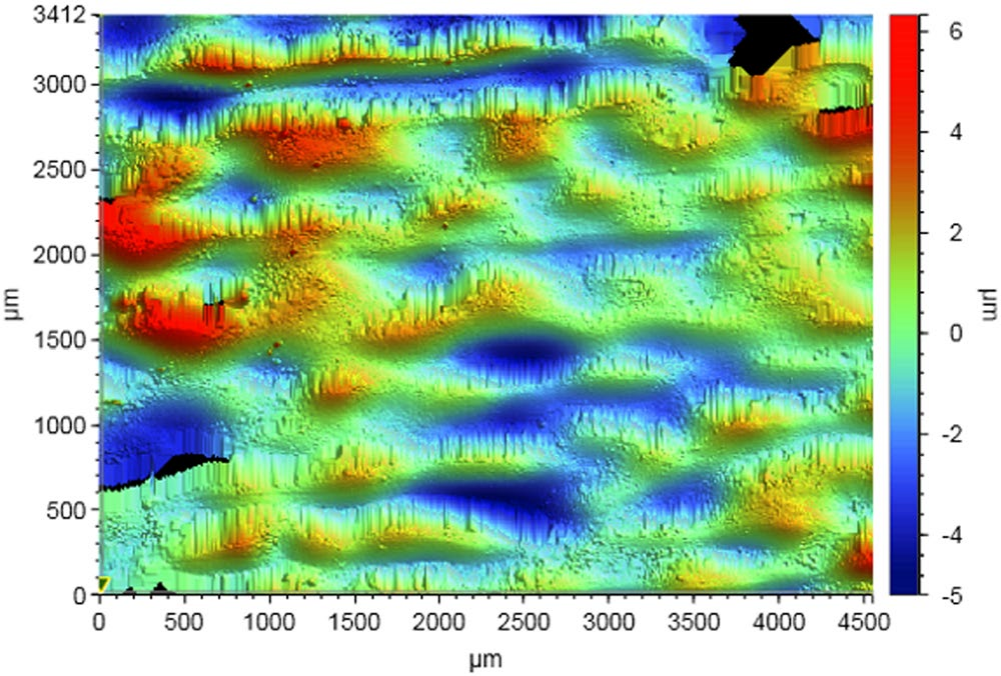
Surface topography of the VeroClear sample.

Figure 3 shows the surface topography of the 3D printed VeroClear sample. The surface of the printed parts was undulating, an inherent nature of the material jetted parts^12^. Due to the wavy nature, the surface roughness (Ra) of the printed VeroClear sample was 2.7 ± 2.3 µm. Since the focus of the study was to print conductive tracks on an as-fabricated inkjet printed surface to enable MFAM, no surface modification was performed to smoothen the surface.

### Print height optimisation

**Figure 4:**
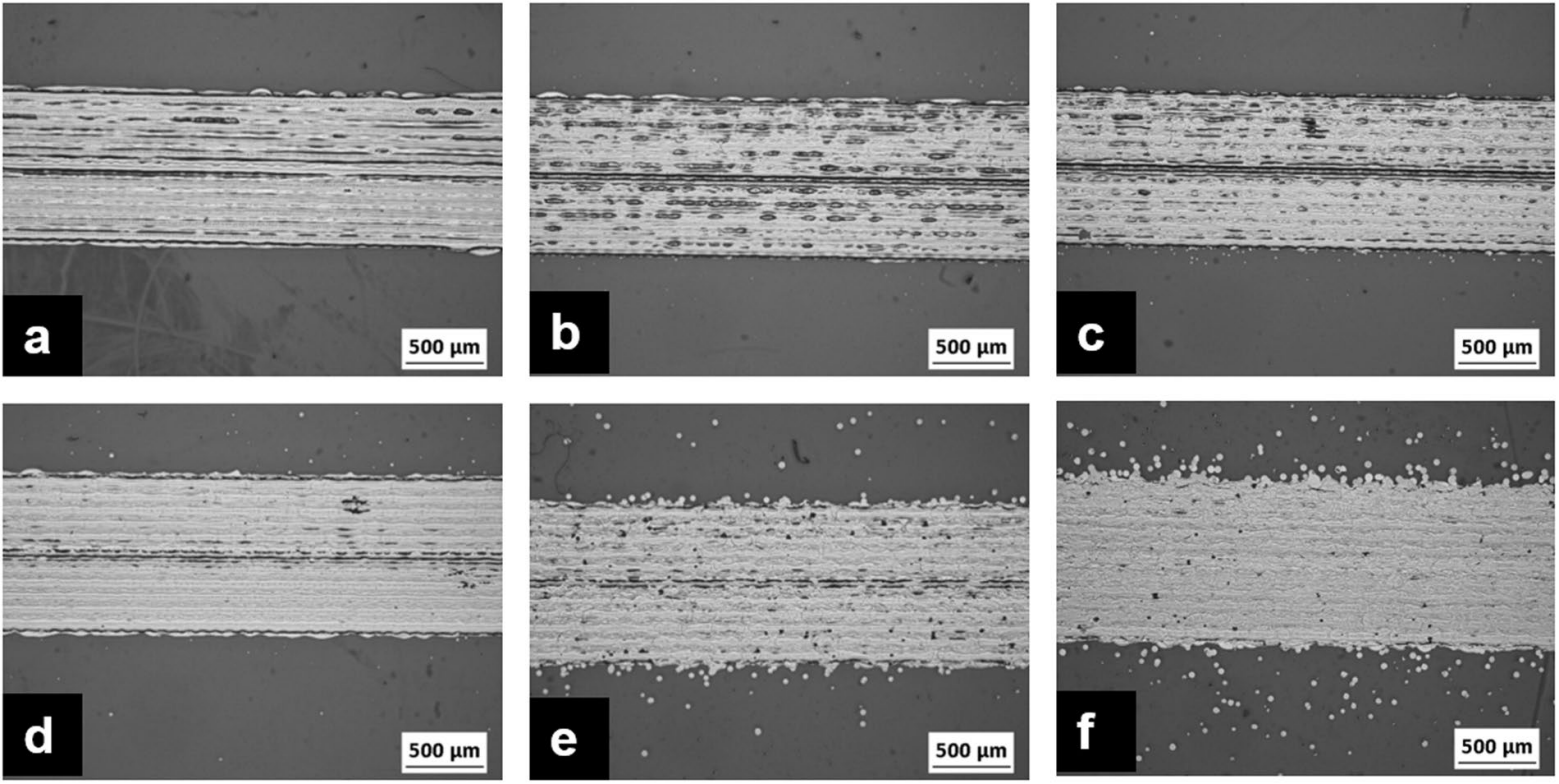
Surface morphology of the tracks printed and sintered on glass slides at various print heights **(a)** 1 mm, **(b)** 2 mm, **(c)** 3 mm, **(d)** 4 mm, **(e)** 5 mm and **(f)** 6 mm.

**Figure 5:**
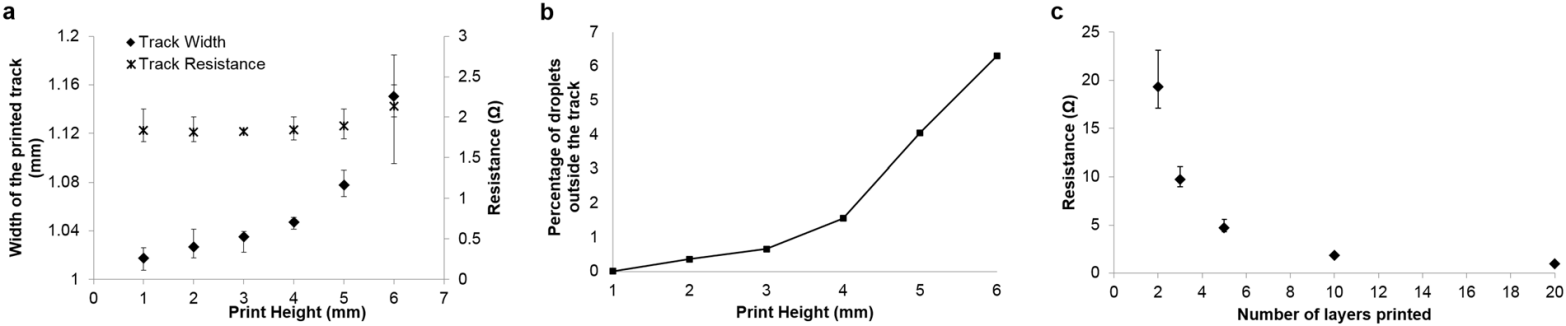
Change in the width of the printed track and electrical resistance of the printed tracks as a function of print height **(a)**; Percentage of droplets deposited away from the track **(b)**; Electrical resistance of the printed tracks for different number of layers **(c)**.

Experiments were performed to optimise the maximum print height from which the AgNP inks could be jetted without creating splashing of the ink droplets upon deposition. Figure 4 depicts optical microscopic images of the tracks printed on a glass slide from 1– 6 mm print height with an increment of 1 mm. It can be noted from the figure that with the increase in print height, satellite formation is more pronounced. These images of the tracks in Fig. 4 were image processed to calculate the track width and percentage of droplets deposited away from the printed track with respect to varying print heights. Figure 5a illustrates the width of the tracks printed at various print heights and the corresponding electrical resistance. Figure 5b shows the percentage of droplets deposited away from the printed track with respect to varying print heights. It is evident from the presented results that with the increase in print height particularly above 3 mm, there is a significant increase in the drifting of the AgNP ink droplets from the actual pattern. In comparison to the track printed with 1 mm print height, a 6% and 13% increase in the track width for print heights 5 mm and 6 mm respectively was witnessed. This was mainly due to the drifted 4% and 6% droplets away from the main track for 5 mm and 6 mm print heights. As a result, with the increasing print height, the cross sectional area of the track is reduced and thus increased the electrical resistance of the printed tracks.

One of the main advantages of inkjet compared to other traditional methods is the precise position of droplets and resolution. Despite no significant increase in resistance being observed for tracks printed up to a 5 mm print height, there is a 4% drift in the ink droplets. These droplets scattered away from its trajectory will affect the resolution and reduce the amount of material in the printed pattern, especially when printing on an angular surfaces which is in focus of this study. Since the splashing of droplets and electrical resistance was low for tracks printed with a print height of 4 mm, this print height was chosen for the further studies.

### Print layer optimisation

The effect of print layers on the conductivity was obtained by studying the electrical resistance of the printed tracks at 1, 2, 5, 10 and 20 layers. Tracks with a single printed layer showed a higher resistance (252.5 Ω) compared to multiple layers, since the track formation was incomplete. Hence the result was not included in the plot in Fig. 5c. It can be noted from the figure that the electrical resistance drops with the increase in the number of layers. The deviation of the data from the mean was also observed to decrease with an increase in the number of layers. The resistance achieved after 10 layers was noted to be sufficient to understand the effect of slope on the conductivity and there was no further benefit in adding more layers. Hence 10 layers of silver was chosen as the optimised number of layers required for this study.

### Thermal stability

**Figure 6:**
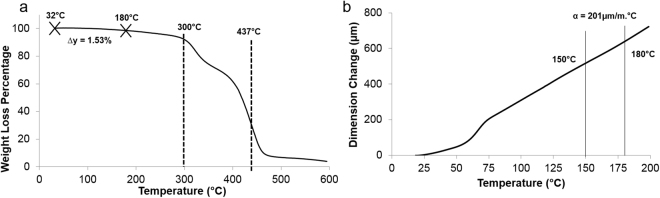
Thermogravimetric **(a)** and thermal expansion **(b)** curve for VeroClear sample printed in Objet Connex3 260.

TGA was performed to understand the stability of the printed VeroClear sample when exposed to the AgNP sintering temperature of ~150 °C to 180 °C. Figure 6a shows the TGA plot for the printed VeroClear sample. The material showed a significant weight loss between 320 °C and 437 °C. From 30 °C to the sintering temperature regime, 1.5% weight loss was observed. The TMA analysis (Fig. 6b) revealed that the VeroClear sample exhibited a thermal expansion co-efficient (α) of 201 ± 0.2 µm/m °C in the range 150 °C to 180 °C.

### Surface morphology of conductive tracks printed on VeroClear substrate

**Figure 7:**
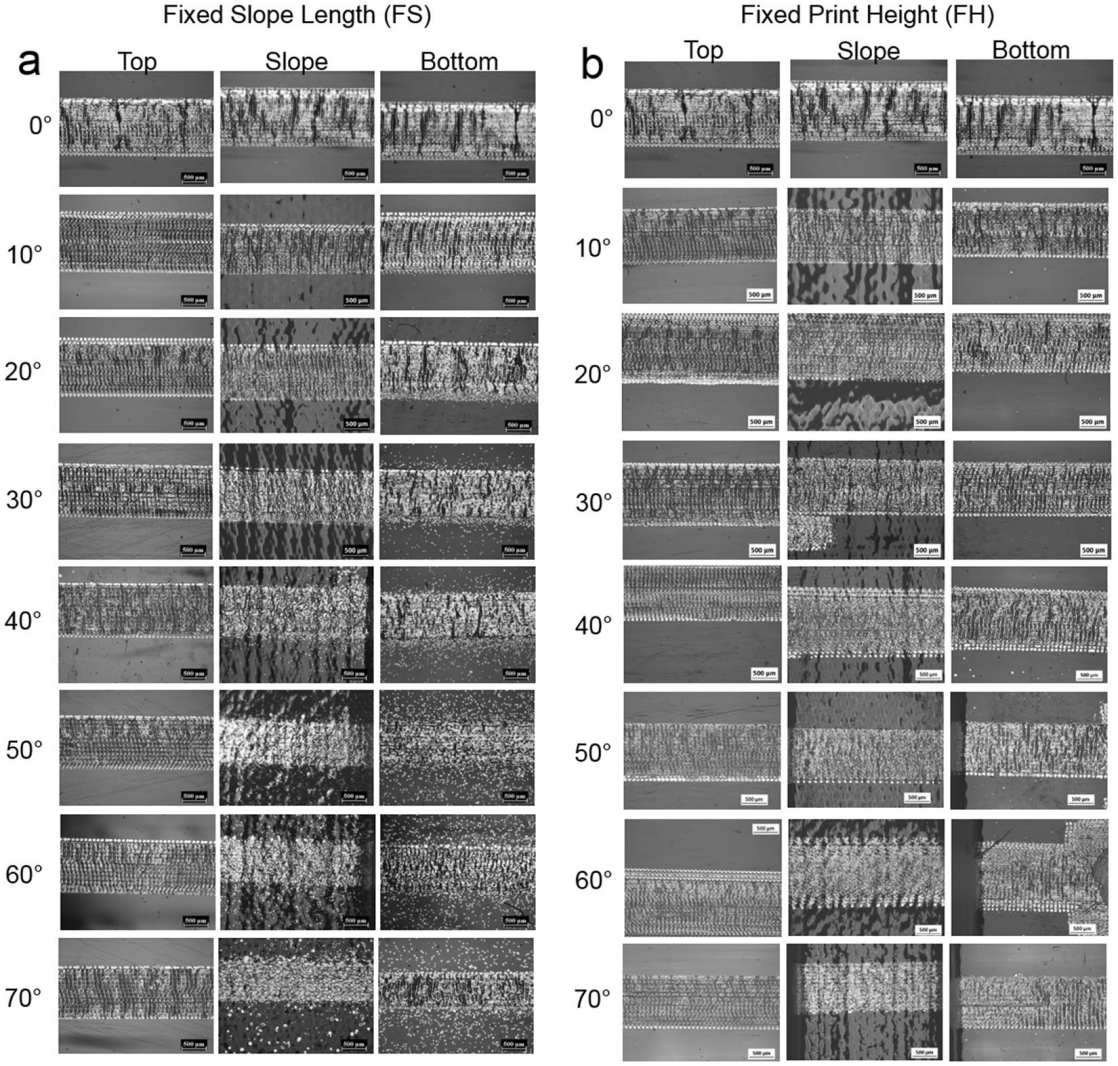
Surface morphology of printed and sintered AgNP tracks on the VeroClear surface for angles 0°–70° **(a)** fixed slope - FS and **(b)** fixed height–FH.

Surface morphology of the tracks printed on FS and FH samples with various angles have been shown in Fig. 7. Surface morphology of the tracks for angles 0° to 70° with 5° increments is available in the Supplementary Information S1. For the FS sample, the tracks printed on the top surface showed uniformity for all angles when compared to the slopes and the bottom areas of the part. This was due to the constant print height (1 mm) at the top but not in the bottom surface of the samples since the print height changed depending on the slope angle (Figure S2, Supplementary Information). For angles above 45°, as the slope was steep and the distance from the print head varied, the tracks were not printed precisely and continuously in the slope region. As mentioned in previously, the drifting of the droplets were more significant with the increase in the print height.

In case of the FH sample, there was minimal distortion in the printed tracks on the top and bottom surfaces since the print height was constant. However, the pattern on the slope of the samples above 65° showed some order of discontinuity due to the steep angle. In addition, the presence of scattered droplets were less significant in the FH samples than the FS sample. As the slope length of the samples decreased with increasing angles, an undulating surface topography was more prominent and a stair-like profile was observed on the substrates with low angles (Figure S3, Supplementary Information). These undulating behaviour is due to the layer-by-layer printing of substrates.

**Figure 8:**
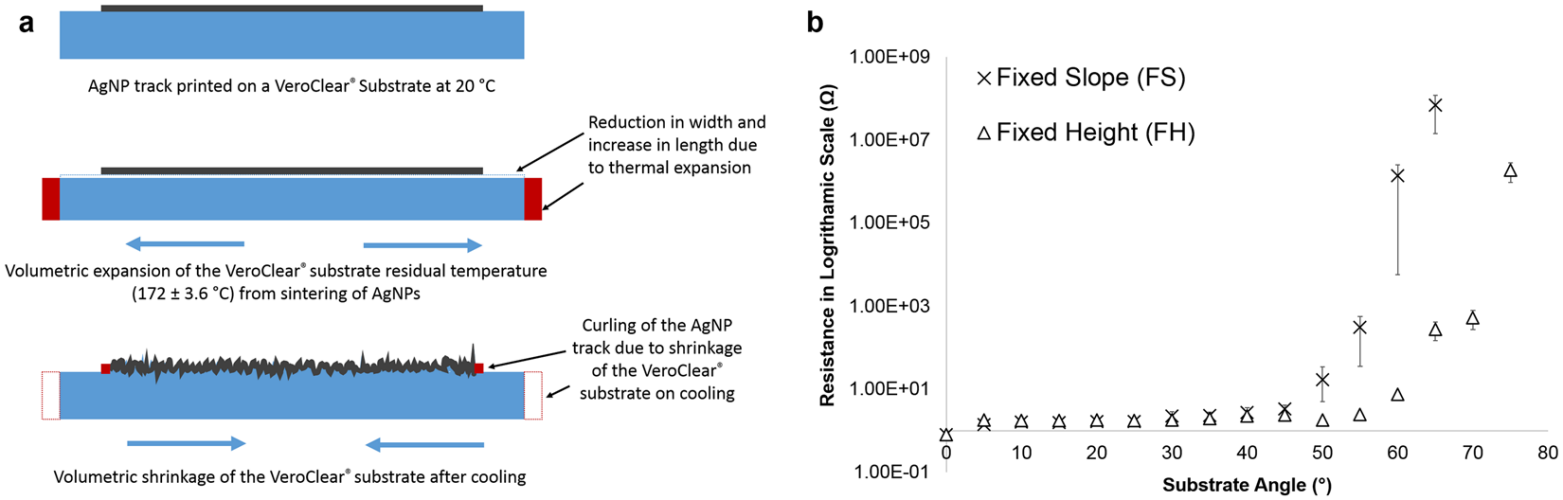
Schematic representation of the thermal expansion behaviour observed on the VeroClear substrate. **(a)** Electrical resistance measured for the printed AgNP tracks on fixed slope (FS) and Fixed Height (FH) samples **(b)**.

A number of crack-like regions on all printed tracks for both FS and FH samples were observed irrespective of the location of the track (top/slope/bottom). These crack-like regions are believed to have occurred due to the thermal expansion of the polymeric substrate when exposed the sintering temperature above 170 °C. Although the first layer of AgNP ink was printed on the VeroClear substrate at 20 °C, after sintering the AgNPs under the IR lamp, the surface temperature of the VeroClear was noted raise to 172 ± 3.6 °C. Based on the thermal expansion co-efficient (α) of 201 ± 0.2 µm/m. °C measured for VeroClear at this temperature regime, the 70 mm long sample is expected to expand ~14 µm. Hence the rest of the 9 layers of the AgNP tracks were printed on this expanded surface. Once the printing is complete and the substrate is allowed to return to the room temperature (20 °C), both the silver and the VeroClear is expected to shrink. As a result of this shrinkage, the AgNP track printed on VeroClear substrate was observed to curl with a reduction in the length of the track. This phenomenon has been schematically shown in Fig. 8a. Thus the crack-like protrusions which are essentially due to the shrinkage of the material were observed on the AgNP tracks printed on the VeroClear substrates. It should be noted that any discontinuity in the AgNP tracks due to these protrusions will possibly impact the electrical conductivity.

### Electrical resistance

Despite the attempts to obtain cross-sectional area of the complete track using a surface profilometer, it was difficult due to the scattering of ink droplets, surface profile of the polymer substrate on the slope and discontinuities in the conductive tracks printed on the slope and bottom regions of the samples. Since the cross-sectional area is crucial for the calculating the resistivity and due to the challenges mentioned above, electrical resistance of the printed tracks were measured and reported. The electrical resistance measured for the AgNP tracks printed on VeroClear substrate for FS and FH samples with varied angle is shown in Fig. 8b. As it can be noted from the results, for the FS samples, the increase in the angle increased the print height at the angular and the bottom surfaces of the samples. After 15°, the print height was observed to be more than 3 mm (Figure S1, Supporting Information). As a consequence, due to this varying print heights with respect to increasing angle, the misplacement and or drifting of the droplets away from the trajectory was more dominant and affected the resolution of the printed tracks. These scattered droplets away from the track are known to reduce the cross sectional area of the tracks, contributing to the increase in the electrical resistance. The electrical resistance remained below 3.5 Ω for the FS and 2.3 Ω for the FH samples up to 45°. After this point, a sharp increase (five times) in the resistance of the FS samples was witnessed, which is attributed to the less cross sectional area due to significant scattering of ink droplets. In terms of the FH sample, the resistance remained low (>2.5 Ω) until 55°, after which it started to increase to 3 times the value, leading on to a sharp increase in the resistance. As there was no/fewer scattered droplets in the FH samples, the increase in the resistance is attributed to the steep angles above 55°. At these steep angles, the ability of the ink droplets to fall precisely on to the track and forming a continuous film is difficult. Also, there is a possibility for some regions of the tracks in the angular surface can be thicker than the other due to flow of the ink. In addition to this, since the underlying part was printed layer-by-layer, the undulating morphology of the surface is more prominent in the angular regions, possibly restricting the droplets to merge with each other to form a continuous track. Furthermore, in this study, the print resolution (500 dpi) of the pattern was the same for both the planar and angular regions. In consequence, the number of droplets used for the planar region will be insufficient for the angular region since the droplets required to cover diagonal length in angular surfaces will be more than the planar surfaces (Figure S4, Supplementary Information). As a result of insufficient droplets in the angular regions, the cross sectional area will be less and it can contribute to the electrical resistance of the printed AgNP tracks.

## Conclusion

This study shows the feasibility of printing conductive tracks on angular surfaces using inkjet printing. Print height is key in determining the print resolution and hence it has to be fixed to a certain height to obtain a uniform and less resistive tracks. The optimal angle range to obtain the least resistive tracks was using fixed height approach was 50–60°. This approach of printing conductive tracks at an angle will help to reach the ‘Z’ height faster than printing conductive tracks on the same plane. Further, this approach will potentially reduce the exposure of dielectric material to high temperatures and the degradation of the material.

## Methods

### Materials

RGD810 VeroClear (purchased from SYS, UK) was used as the substrate material to fabricate parts with varying angles for the FS and FH samples. Objet support SUP705 (SYS, UK) was used as the supporting material. Silverjet DGP-40LT-15C AgNP ink (Advanced Nano Products, Korea) with 38.85 wt% AgNP in Triethylene Glycol Monoethyl Ether (TGME) was used to print conductive tracks. Soda lime glass substrates were used for the printing of reference conductive tracks in order to understand the impact of the number of layers and the print height on electrical resistance without the presence of polymer. Isopropyl alcohol ≤99.5% (Sigma Aldrich, UK) was used to clean the glass slides and VeroClear samples before printing. Silver conductive paint and Kapton tape was supplied by RS Components, UK. Silver conductive paint was purchased from RS Components, UK.

### Design approach

In order to print substrates with angles ranging from 0°–80° (with 5° increments), two different approaches, Fixed Slope (FS) and Fixed Height (FH) were adopted as shown in Fig. 1. For the FS sample, the slope distance was fixed and the height of the sample was increased; whereas for the FH sample, the print height was fixed and as a result the slope length changed for every angle.

### Design

3D models were designed using Magics 19.01 (Materialise) computer aided design (CAD) software. The dimensions of the FS and the FH samples are shown in Fig. 1. Angles ranging from 0° to 80° (in increments of 5°) were designed for both sample types. The designed samples were aligned to the build platform for fabrication. Five cylindrical samples of diameter 10 mm and height 20 mm for the thermomechanical analysis (TMA) was also designed. Three equally spaced (5 mm) tracks for printing AgNPs with 35 mm length, 1 mm width along with a contact pad on either end (2.5 mm × 2.5 mm) was designed using GIMP (The General Image Manipulation Program) software with a resolution of 500 dpi (dots per inch). The pattern was saved as a bitmap (.bmp) file.

### 3D-printing

#### Substrate

A Stratasys Objet Connex3 260 3D inkjet printer was used to fabricate the FS and FH samples. Briefly, the designed patterns (0°–80° with increments of 5°) were aligned to the platform. VeroClear was used to build the samples. Print heads and the build platform were cleaned using IPA. A “glossy” finish was selected for all the samples. The parts were printed parallel to the printing direction to minimise the effect of ridges on the surface. Post-fabrication, the parts were removed from the build platform and supporting structures were also removed. TMA samples were also printed using the same print conditions in the Objet machine.

#### Conductive tracks

Conductive silver tracks were printed using a bespoke PixDro JETx machine. Each FS and FH samples were rinsed with IPA and dried before placing onto the build platform. Since the build platform of the JETx is maintained at 20 °C, the samples were positioned above the platform using two rectangular ceramic blocks (5 mm thick) in order to prevent the IR heating system of the JETx interfering with the cooling system of the build platform. A glass slide was placed above the ceramic blocks and secured with a section of Kapton tape. A VeroClear sample with 0° slope was placed on the glass slide and secured with Kapton tape. The pattern was then aligned to print on the VeroClear sample. The AgNP ink was printed from a print height of 1 mm using Spectra SE 128 print head (Fujifilm, USA) translating at a speed of 300 mm/s. For optimising the print height, the height of print head from the sample surface was varied from 1 mm–6 mm with 1 mm increments. After printing each layer, the printed ink was sintered using an in-built IR lamp (Heraeus 4114; 2KW). A previously optimised sintering condition was used for sintering the AgNPs^21^. Briefly, the IR lamp was set at 50% intensity, the height of the substrate from the IR lamp was 7 mm; movement of platform under the IR lamp was at the rate of 5 mm/s with 10 passes of IR per printed layer. The build was stopped after the completion of 4^th^ and 7^th^ layer to examine if the VeroClear sample has lifted-off from the glass slide due to thermal expansion. This check was performed to avoid collision of the samples with the print head. Once the printing was complete, the sample was allowed to cool at room temperature (20 °C). An RS1327K (RS Components, UK) IR thermometer with ±2 °C accuracy was used to obtain the temperature of the VeroClear surface after sintering the AgNPs. A similar procedure was followed to print conductive tracks on all FS and FH samples.

### Characterisation

#### Surface morphology and topography

Droplet size and surface morphology of the printed samples were obtained using a Nikon Eclipse (LV100ND) optical microscope. The sizes of 15 droplets were measured and averaged. The VeroClear samples were tilted to an angle so that the track appeared flat and the morphology of the printed silver tracks on the slopes was imaged. Surface topography of the VeroClear samples was obtained using a Bruker white light interferometer with 2.5x optical lens.

#### Image processing

The optical microscopic images obtained for AgNP tracks printed and sintered on a glass substrate from varying print height was image processed using MATLAB in order to study the percentage of droplets scattered around the printed tracks. The images used for the analysis were obtained with same magnification. Various threshold values were used and superimposed on the original image to obtain a visually true representation of the image. The obtained image was processed for the percentage of black and white areas to obtain the percentage of droplets away from the tracks. It should be noted that this analysis is using a 2D image and hence there is a possibility for this analysis to be affected by the overlapped ink droplets.

#### Thermal stability

Thermogravimetric analysis (TGA) of the printed VeroClear sample was obtained using Perkin Elmer TGA 4000 Thermogravimetric Analyzer to study the decomposition temperature of the printed VeroClear samples. A small amount (~10 mg) of the printed VeroClear sample cut from a previously fabricated sample using the same process conditions and used for the analysis. The material was heated from 30 °C to 600 °C at the rate of 10 °C/min. Percentage weight loss with respect to temperature was obtained. TMA of the printed VeroClear sample was obtained using TMA Q400 (TA Instruments, UK). A macro expansion probe was used during the study. The flow rate of nitrogen was 100 mL/min. A preload force of 0.01 N was used. The sample was heated from 20 °C to 200 °C at the rate of 5 °C/min. The static applied force during the temperature ramp was 0.02 N. The temperature precision of the equipment is ±1 °C and the measurement precision is ±0.1% according to the manufacturer’s guidance.

#### Electrical properties

Resistance of the printed and sintered Ag tracks were obtained using a Hameg LCR high precision meter (HM 8018; Rhode and Schwarz, UK). A small drop of conductive silver paint was placed on the printed contact pads in the silver tracks in order to avoid scratching of the tracks by the probes during measurement. The samples with silver paste were dried in a convection oven at 75 °C for 15 mins to evaporate solvents in the paste. Resistance offered by the silver tracks was obtained from three tracks and the results were averaged. The reported ± value is the range from the mean.

## Electronic supplementary material

Supplementary Information
