# Supplementary material for: Optimisation of Substrate Angles for Multi-material and Multi-functional Inkjet Printing
Source: Sci Rep. 2018 Jun 13;8:9030. doi: 10.1038/s41598-018-27311-6 (PMC5998081; doi:10.1038/s41598-018-27311-6)
Supplement: Supplementary file 1 — Supplementary Information [file 41598_2018_27311_MOESM1_ESM.pdf]

Supplementary Information

Optimisation of Substrate Angles for Multi-material and Multi-functional Inkjet Printing

Jayasheelan Vaithilingam*, Ehab Saleh, Ricky D. Wildman, Richard J.M. Hague and Christopher J. Tuck

Centre for Additive Manufacturing, Faculty of Engineering, University of Nottingham, Nottingham, UK, NG7 2RD.

Supplementary Information

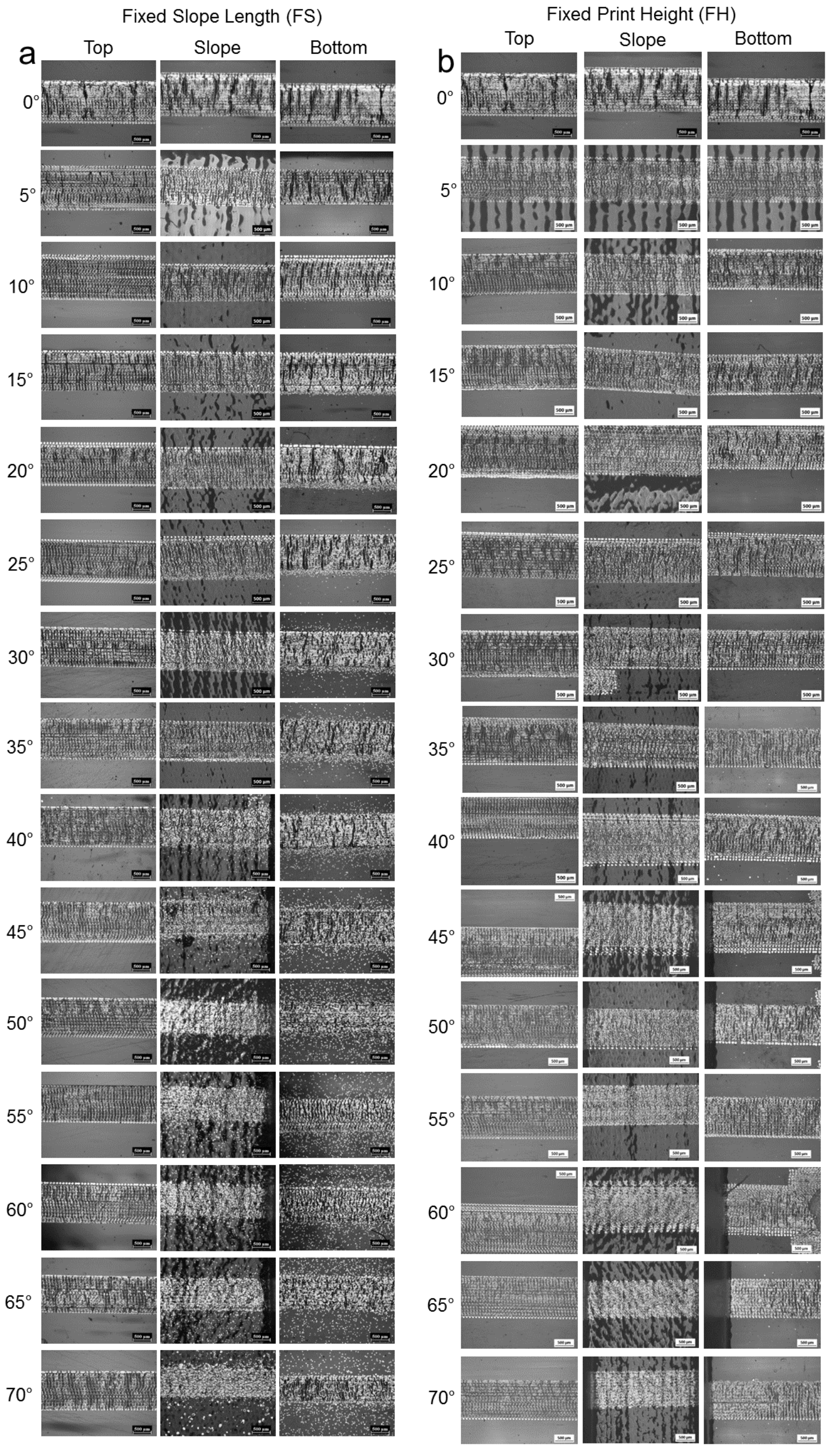

Figure S1 Surface morphology of printed and sintered AgNP tracks on the VeroClear surface for angles 0° - 70° (a) fixed slope - FS and (b) fixed height - FH

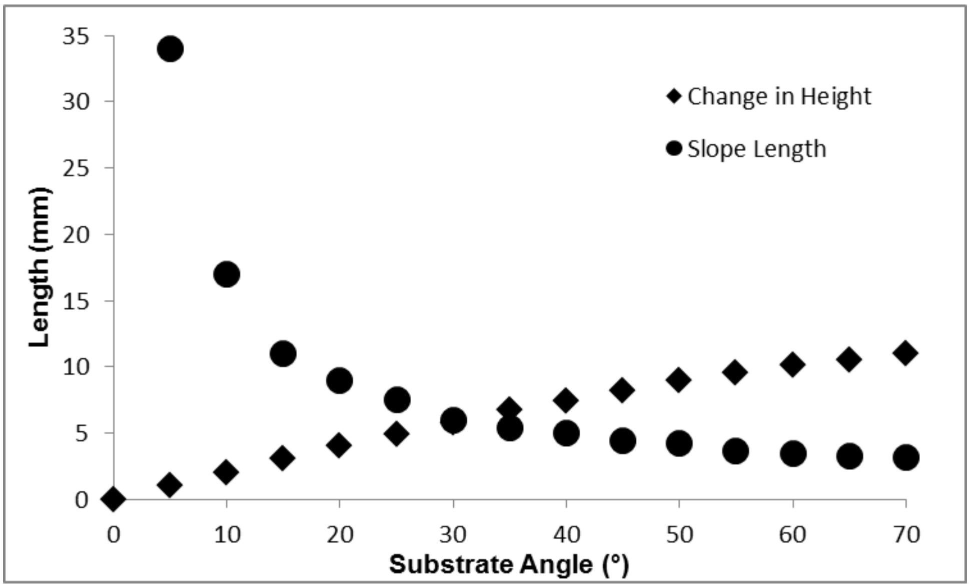

Figure S2 Height and slope length as a function of substrate angle

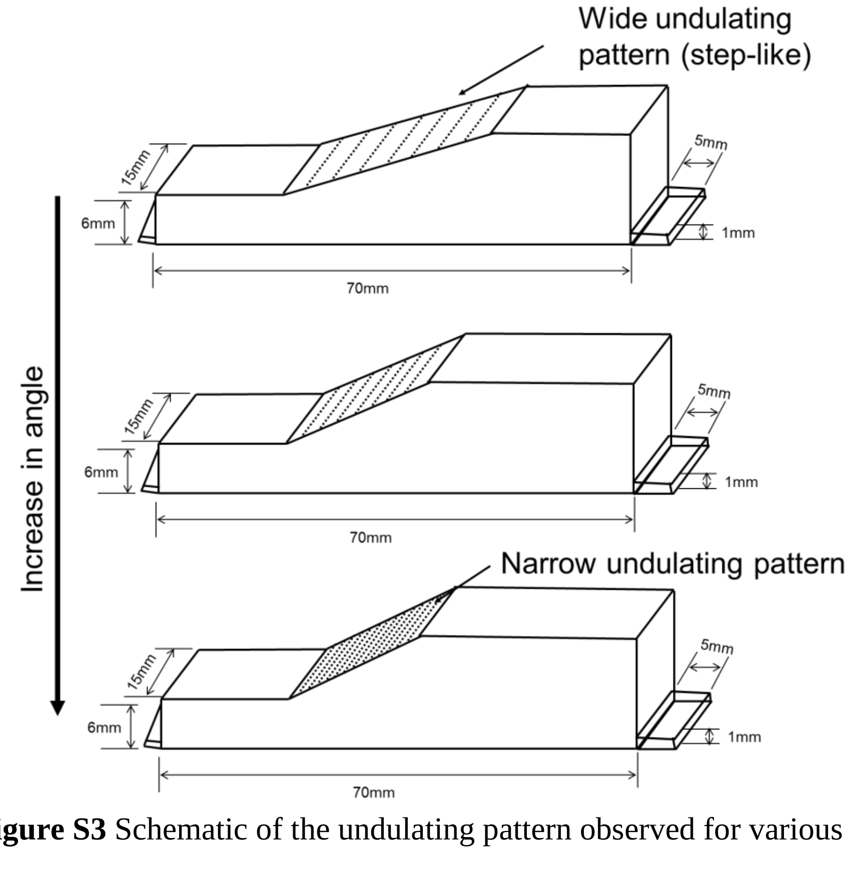

Figure S3 Schematic of the undulating pattern observed for various angles

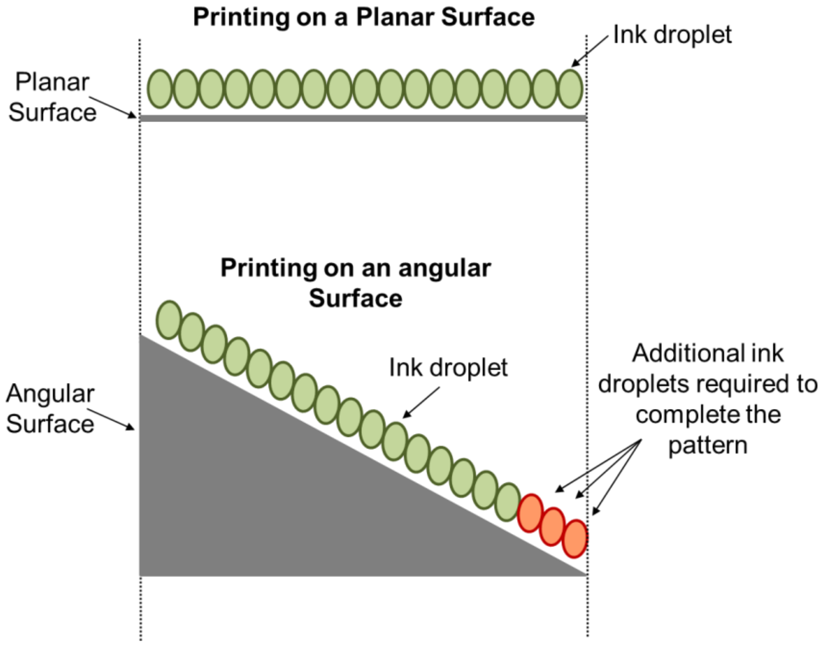

Figure S4 Schematic showing more droplets are required for an angular surface than a planar surface
